# Improving care for older patients living with frailty: a collaborative approach to creating the DANFRAIL quality database

**DOI:** 10.1007/s41999-025-01395-6

**Published:** 2026-01-12

**Authors:** Lone Winther Lietzen, Søren Kabell Nissen, Karen Andersen-Ranberg, Mette Theil, Louise Moeldrup Nielsen, Sacha Methe Elbeck, Erik Riiskjær, Anne Mette Falstie-Jensen, Thomas Johannesson Hjelholt

**Affiliations:** 1 Department of Geriatrics, Aarhus University Hospital, Aarhus, Denmark; 2 Department of Clinical Medicine, Aarhus University, Aarhus, Denmark; 3 Department of Emergency Medicine, Odense University Hospital, Odense, Denmark; 4 University of Southern Denmark, Odense, Denmark; 5 The Danish Dietetic Association, Copenhagen, Denmark; 6 Research Center for Care and Rehabilitation, VIA University College, Aarhus, Denmark; 7 Local Government Denmark, Copenhagen, Denmark; 8 Aalborg Municipality, Denmark; 9 Patient and Relatives’ Representative in the DANFRAIL Steering Committee, Aarhus, Denmark; 10 The Danish Healthcare Quality Institute (DHQI), Aarhus, Denmark; 11 Department of Geriatrics, Odense University Hospital, Odense, Denmark

**Keywords:** Co-design healthcare, Frailty, Older, Quality indicators, Indicator set, Comprehensive geriatric assessment

## Abstract

**Background:**

Older patients living with frailty are particularly vulnerable to adverse outcomes during hospitalization and transitions in care. Comprehensive Geriatric Assessment (CGA) has been shown to improve outcomes for these patients. To address the increasing demand for geriatric methodology and improve care quality, the nationwide quality database DANFRAIL was established with a cross-sectoral and interdisciplinary focus, first of its kind worldwide.

**Methods:**

DANFRAIL aims to monitor and enhance quality of care for older patients with frailty across healthcare settings in Denmark. Thus, development involved a co-creation process with expert clinicians and patient representatives from all sectors, guided by a specialist team from the Danish Healthcare Quality Institute. The indicator set, derived from CGA domains, was refined through consensus meetings and public consultation.

**Results:**

The initial indicator set includes 6 process indicators and 2 result indicators, focusing on key aspects of care for older patients living with frailty. The public consultation revealed concerns about implementation, workload, registration requirements, and data integration across sectors. Adjustments were made to address these issues and ensure the feasibility of data collection and use.

**Conclusion:**

DANFRAIL represents a significant step towards improving the quality of care for older patients living with frailty in Denmark. Grounded in the principles of CGA, the database offers a structured, data-driven framework to monitor and enhance care delivery across sectors. By supporting continuous quality improvement and better health outcomes, DANFRAIL sets a national standard for caring for older people in vulnerable situations and may serve as an exemplary approach for international adoption.

## Background

Older patients living with frailty are vulnerable to hospitalization and transitional care, as they possess limited intrinsic resources to overcome stressors and complex health issues [1]. Thus, specialized interdisciplinary and cross-sectoral approaches are needed to provide high-quality care [2]. Despite the existence of well-documented interventions, healthcare systems still struggle to fulfill these patients' comprehensive needs [3]. An increasing pressure on most western healthcare systems is currently observed, possibly due to a demographic shift toward an aging population [4], workforce shortages, insufficient digitalization, inappropriate treatment [5], unrecognized overtreatment [6], and limited hospital bed capacity. These challenges are foreseen to intensify in the coming years and calls for a structured approach to ensure sufficient quality across the entire healthcare system.

Comprehensive Geriatric Assessment (CGA) [7] is a multidimensional diagnostic and therapeutic process aiming at identifying the medical, functional, mental, and social capabilities and limitations of an older person living with frailty. It furthermore facilitates dialog with the patient to help prioritize and manage identified deficiencies. When delivered by a multi-disciplinary team, the CGA approach has been found to improve the likelihood that an older person living with frailty will be alive and living in their own home after hospitalization [2]. The CGA also ensures the delivery of person-centered healthcare in a coordination manner, ensuring cross-sectoral continuity, which is considered a cornerstone of high-quality healthcare [8].

In response to the described challenges and to proactively address the growing need for geriatric methodology in Denmark, the Danish Geriatrics Society (DGS) applied for the establishment of a national clinical database aiming to monitor variations in the care of older people living with frailty and to support data driven quality improvement initiatives in collaboration with the nationwide Danish Healthcare Quality Institute (DHQI). In 2022, the application was approved and prioritized by the DHQI steering committee, which subsequently initiated a co-creation process with clinicians and patient relatives.

The aim of the database is to monitor and reduce unwarranted variation in treatment and care decisions for all older patients living with frailty in Denmark—regardless of where they interact with the healthcare system. To capture a balanced expression of clinical quality within a complex and multidimensional healthcare system, the database was designed to reflect properties within multiple domains [9]. Therefore, CGA was selected as the conceptual framework to define these domains, ensuring a balanced, multidisciplinary, and patient-centered approach to quality measurement. In 2023, the proposed indicator set underwent public consultation prior to nationwide implementation.

This article presents the development process leading to the first indicator set, key inputs from the public consultation, and the rationale for the multidimensional design supporting the database’s core objective.

## Methods

### Setting

Healthcare in Denmark is tax-funded and offers universal healthcare and social support to promote health and social equity [10]. The system operates across three political and administrative levels:

1) The state is responsible for overall regulatory and supervisory functions.

2) The five regions (reduced to four from 1 January 2026) are responsible for organizing and operating healthcare services, including hospitals, general practice, and emergency care.

3) The 98 municipalities are responsible for providing a range of primary health and social services, including home care and rehabilitation [10].

Due to a unique personal identification number (CPR number) that is assigned at birth or immigration, it is possible to link across all national administrative and clinical registries and have complete follow-up. Despite this, inadequate information-sharing and differences in digital infrastructure challenge coordination and care across sectors and disciplines [11].

### Clinical quality databases

Based upon the model for improvement [12], the DHQI measure quality within approximately 85 disease or symptom areas reflecting important healthcare topics in Denmark in order to support quality improvement in the whole course of disease for the patients [13] Each area involve clinicians and patient representatives and use evidence-based indicator monitoring following Donabedian's framework [14] and Deming's Plan-Do-Study-Act circle [15, 16].

### Co-production in a learning healthcare system

The DHQI's work is based on the principles of the Learning Healthcare System [17, 18], which focuses on optimizing outcomes with data-driven insights to inform decision-making. They also emphasize continuous learning and adaptation of ways to improve patient care in collaboration with patients, clinicians, and healthcare leaders.

Workshop and consensus meetings.

Initially, the DGS board invited its members, consisting of physicians specialized in geriatric medicine, to participate in a series of online meetings to share their thoughts on a new database and to reflect on relevant issues that, based on their clinical experience, would be relevant to monitor in a quality database. Condensing the output, the evidence-based framework CGA was chosen as the conceptual foundation, as it provides a holistic and systematic approach to managing care for this complex patient population.

**Fig. 1:**
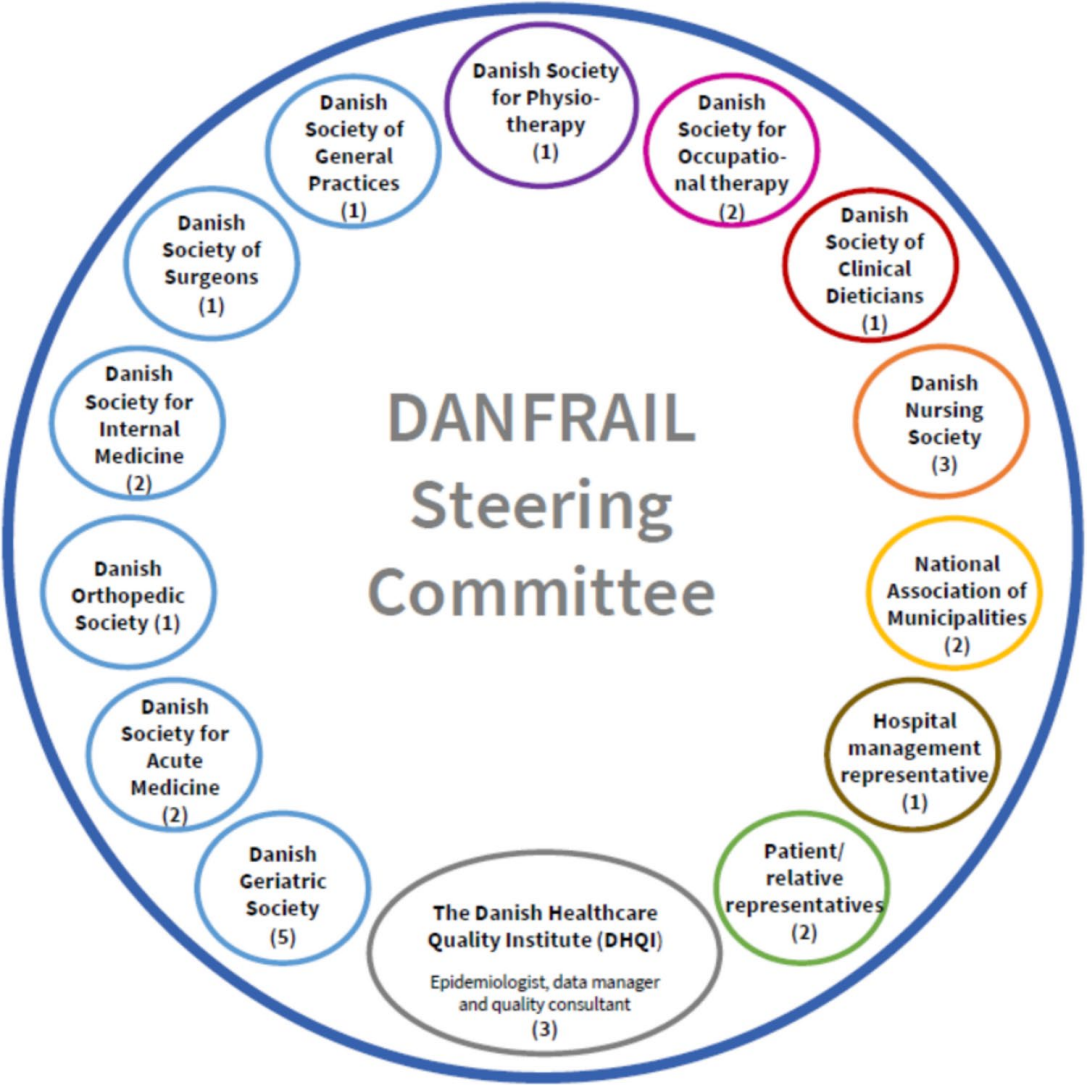
Composition of the DANFRAIL Steering Committee. Members represent a broad range of professional affiliations across all organizational levels of the Danish healthcare system. This inclusive structure ensures local anchoring and sectoral relevance. The chair is currently shared among representatives from the Danish Geriatric Society, the Danish Society for Acute Medicine, and the National Association of Municipalities (Local Government Denmark). The (number) indicate the number of members on the committee

Based on the overarching purpose of the database, namely to improve and harmonize the quality of care for older patients with frailty regardless of where they meet the healthcare system, the DHQI and DGS conducted a workshop with participants representing various stakeholders such as patients' associations, other medical specialty societies, municipalities, nurses, dietitians, physiotherapists, and occupational therapists. The meeting resulted in the identification of key organizations and stakeholders to be included in the database steering committee. Professional societies and organizations accepted invitations to participate in an appointed steering committee. To represent the perspectives of patients and relatives, the DaneAge Association [19] and a relatives’ representative were invited. Three DHQI-designated representatives, an epidemiologist, a statistician, and a contact person, led the process. For a full overview of the steering committee, see Fig. 1.

### Considerations of population delineation

Assessing the quality of care provided to older patients living with frailty across various points of healthcare contacts represents a substantial challenge that necessitates segmentation into smaller, manageable projects. Following the formation of the database steering committee, a series of decisions was made (described in the following section), informed by group discussions, clinical insights, exploratory data analyses, and findings derived from existing literature.

Across four separate full-day meetings, the definition of an old patient living with frailty was refined along with indicator domains (CGA framework) and patient journey mapping [20]. Multiple virtual meetings were held for each domain to specify evidence-based and obtainable indicators. Subsequently, the agreed indicator set was discussed in two consensus meetings (level of evidence, use of resources, number of indicators, and overall considerations focusing on the patient’s perspective) in March and May 2023.

To identify a target population with a high prevalence of frailty, thereby ensuring relevance for both patients and healthcare staff, a pragmatic inclusion criterion was determined to be patients 80 years or older [21, 22]. Since no current available data driven measure is available early enough in the clinical trajectory to support decision-making, and the precision is limited because parameters required for calculation are not routinely measured prior to in-hospital assessment, a clinical assessment of frailty was prioritized—even though it does take efforts to educate and implement (indicator 1, see below).

To maximize clinical impact on complex patient trajectories, the steering group identified patients with unplanned admissions for acute conditions as the most relevant focus for the initial implementation of the database. Unplanned admission of a patient aged 80 years or older to an Emergency Department comes with an increased risk of adverse outcomes such as delirium, loss of functional capacity, longer length of stay, admission to nursing home, and death [23]. Based on hospital administrative data for unplanned admissions in Emergency Departments in 2021, the database will include approximately 180,000 acute contacts annually in this age category.

Focus on the CGA framework sets the direction for the remaining indicator development.

Unfortunately, it is not yet possible to retrieve routinely collected data from the municipalities or general practice [10], which is why the initial target population is based on hospital-generated data. Furthermore, a structured capture of the patient’s experience of continuity and participation is not available and consequently it is not a part of the initial indicator set.

### Indicator set

**Fig. 2:**
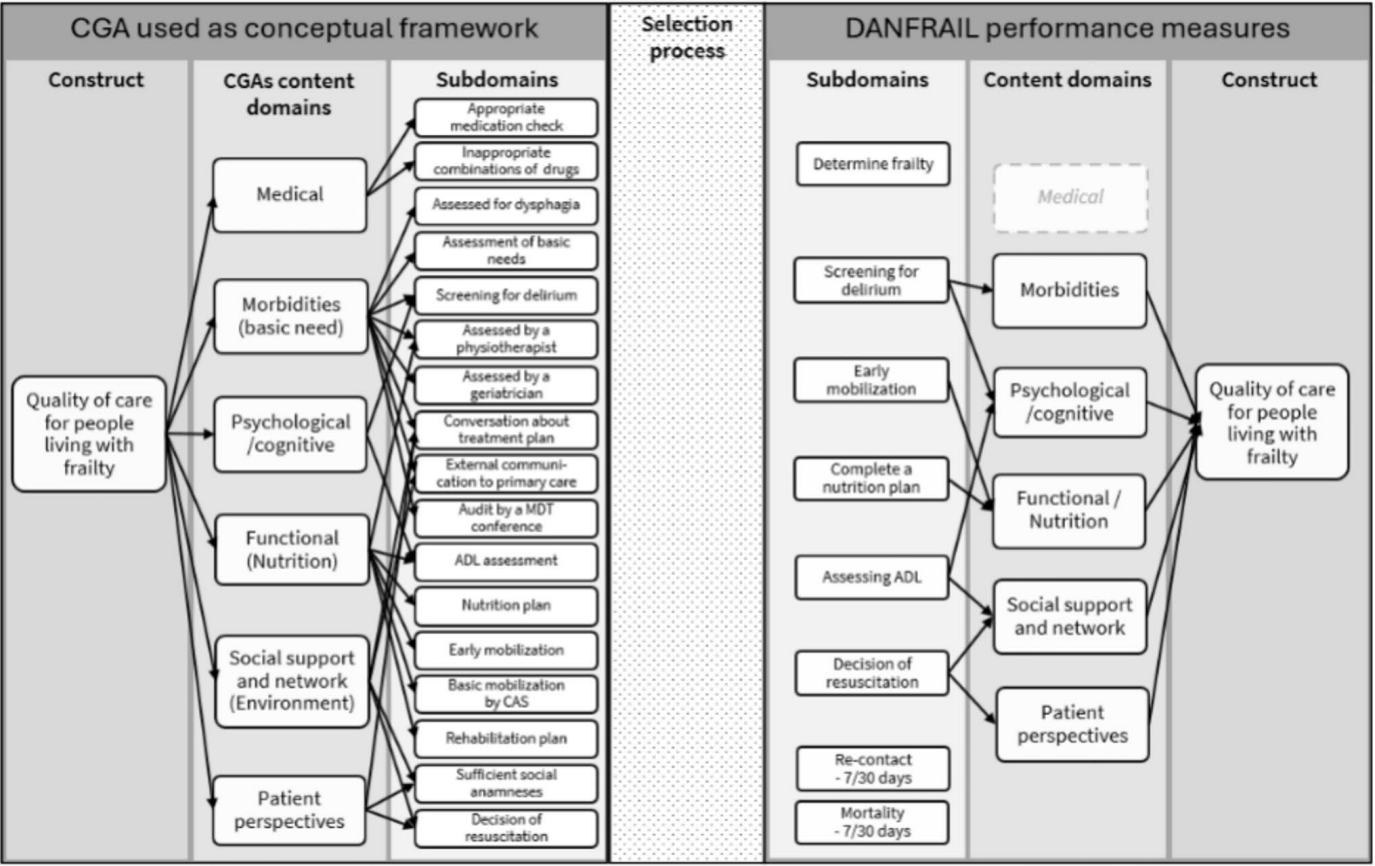
Alignment of the DANFRAIL Indicator Set with CGA Domains. The DANFRAIL indicator set was developed using the Comprehensive Geriatric Assessment (CGA) framework to ensure a holistic and structured approach to evaluating care for older patients living with frailty. The figure illustrates which CGA domains are currently covered by the indicators, as well as areas not yet addressed in the initial version of the database. Figure 2 is inspired by the "lens model" [9]

The indicator set includes 6 process and 2 result indicators (Table 1 and Fig. 2).

**Table 1:** DANFRAIL indicator set

Number	Domain	Wording	Type
1	Determine frailty	The proportion of patients 80 years or older, who are assessed with Clinical Frailty Scale (CFS)	Process
2	Screening for delirium	The proportion of patients with CFS 5–8 who are screened for delirium within 24 h from admission	Process
3a/b	Early Mobilization	The proportion of patients with CFS 5–8 who are mobilized out of bed within 24 h from admission (a) or after surgery (b)	Process
4	Decision of resuscitation	The proportion of patients with CFS 5–8 with admission more than 12 h, who have a do-not-resuscitate evaluation performed within 48 h	Process
5	Complete a nutrition plan	The proportion of patients with CFS 5–8 with admission > 24 h who have a nutrition plan performed	Process
6	Assessing ADL	The proportion of patients with CFS 5–7 with admission > 24 h who have an evaluation of Activities of Daily Living (ADL) prior to discharge	Process
7a/b	Re-contact	The proportion of patients with CFS 5–8 with unplanned hospital re-contact or re-admission within 7 and 30 days	Result
8a/b	Mortality	The proportion of patients with CFS 5–8 who die within 7 and 30 days	Result

The DHQI has made recommendations for the "ideal indicator set", suggesting the number of indicators to be between 5 and 15, and recommending the indicator set to be evaluated at least every three years to incorporate updated evidence and identify potential new areas for quality improvement [24]. The recommendations are in line with a recent editorial advocating for limiting the number of indicators to encourage careful selection, while ensuring regular updates of the evidence and indicators to keep the indicator set relevant for clinical improvement [25].

Indicators will be reported using standardized codes enabling automatized data collection from national registries.

### Identification of patients living with frailty

*Indicator 1:* The proportion of patients 80 years or older, who are assessed with Clinical Frailty Scale (CFS) [26].

Patients are evaluated with a focus on their functional level approximately 2 weeks prior to admission, to capture their habitual level of function. CFS is a 9-level validated judgment-based measure developed to determine the baseline health state and has shown to be easy to implement in acute settings [27]. Among the various frailty measures, the CFS was chosen as it is already widely used in clinical setting, has been translated and validated in Danish, demonstrates acceptable inter-rater reliability across professional disciplines [28], and was assessed by the steering group as the most feasible for implementation in clinical practice as a common language across disciplines and sectors. To support its implementation, an open access case-based online e-learning program was developed.

Based on indicator 1, patients with a CFS of 5 to 8 are included in the database.

*Process indicators:* Corresponding to CGA domains (Fig. 2), the indicators were developed following patient journey mapping and through consensus meetings and adjusted after public consultation (Table 1).

### Evidence report

A basic principle of DHPI is to strengthen the argument for all indicators by an evidence report reviewing evidence for each indicator, based on a systematic literature search. Clinical guidelines were evaluated based on AGREE II [29] and systematic reviews were assessed using ROBIS [30]. The overall evidence was finally graded according to The Oxford Levels of Evidence 2009 [31]. The evidence report is available in Danish at DANFRAIL’s homepage [32].

### Public consultation

During Fall 2023, the initial indicator set and accompanying evidence report was sent out for public hearing. In total, 33 responses were received and evaluated by the steering committee. Hearing parties included the Danish Health Authorities, professional/academic societies, health IT supporters, heads of clinical departments, and an individual physician.

The themes of the responses reflected a broad support for the aim of improving the quality of care for older people living with frailty through the new database. But also, a number of concerns and challenges related to implementation and daily practice were raised. Three important themes were:

1. *Implementation task:* Several responses expressed concern about the extent of the implementation and the practical challenges associated with the introduction of the new database. This included concerns regarding the necessary training of personnel and the integration of new workflows and registrations into an already busy clinical routine.
2. *Timing, relevance, and application of indicators*: There was feedback on the specific indicators included in the dataset, with some parties suggesting adjustments or elaborations to enhance the indicators’ utility and relevance in clinical practice. There was significant interest and support for the use of the Clinical Frailty Scale (CFS) for assessing frailty but also concerns about how and at what point in time this assessment should be implemented in the patient pathway.
3. *Sector transitions and cross-sectoral data exchange*: Several responses called for improved integration and data exchange between sectors, particularly between hospitals and primary care, to enhance the continuity and quality of patient pathways.

Importantly, the representatives of patients and relatives on the steering committee expressed concern regarding the absence of measures for continuity of care and direct patient involvement.

The wording and time specification of the indicators were adjusted according to insight from the public consultation.

### Launch of DANFRAIL

DANFRAIL was officially launched on 1 April 2024 with the first round of data analyses in spring 2025. To support implementation, regional information meetings were held in each of the five regions in spring 2024, fostering dialog between clinicians, local quality improvement core staff, and IT support staff.

## Discussion

The development of the DANFRAIL indicator set followed a structured process rooted in international recommendations for quality improvement in healthcare. Its primary aim is to reduce unwarranted variation in treatment and care across key domains of the frailty syndrome, using evidence-based indicators selected through national consensus. As such, the database is designed to address some of the expected implications associated with demographic development in the coming decades within the framework of a modern healthcare system offering universal access.

### Other databases on frailty

No other national quality databases with mandatory and registry-based data collection have been identified anywhere in the world. However, several databases with similar scope exist; e.g., the National Health and Aging Trends Study (NHATS) from the USA [33] has collected extensive data on older people including frailty-associated domains such as cognitive capacities, physical function, and living conditions. The database is based on self-reporting and interviews and reports descriptive longitudinal data. In Sweden, the SeniorAlert database is a large clinical quality database, but the data collection is based on web registrations and reporting is not mandatory [34]. A widely used platform offering systematic collection of clinical data for quality improvement is the interRAI network that offers indicator sets customized for different clinical settings, e.g., acute care for older patients. However, it still relies on specific testing and reporting procedures and has not been implemented as a mandatory nationwide system in any country to date [35]. Thus, DANFRAIL represents a novel quality improvement initiative by mandating nationwide participation of all acute care hospitals in Denmark and implementing a systematic approach to frailty screening and management based on the CGA framework.

### Strengths

The DANFRAIL database meets the quality standards for establishment of a clinical database recommended by others [9, 25]. They advocate transparency, definition of purpose, and application of conceptual framework to guide the process. Furthermore, they encourage reflection on:

- who will be using the dataset,

- what questions the indicator set is intended to answer,

- what underlying construct is being measured, and

- whether the use of this indicator set may lead to any unintended consequences?

To ensure a transparent and inclusive development process, DANFRAIL was developed through close collaboration between clinicians, representatives of patients and relatives, and guided by the DHQI team. Additionally, a public consultation was carried out, inviting input from a broad range of stakeholders, including public authorities, professional societies, patient organizations, and hospital departments.

The purpose of the database and its conceptual framework was initially outlined in the DGS application, and the adoption of the CGA as the guiding approach was implemented very early in the process. However, as the development progressed, the original purpose was refined to ensure realistic and feasible implementation across diverse hospital departments. The current focus centers on a holistic assessment of quality for patients with acute disease and unplanned hospital admission.

Awareness of different user perspectives must be a key consideration when interpreting the results, as it cannot be assumed that an indicator set will meet the needs of all users equally well reflecting the enormous heterogeneity of older patient’s needs:

DANFRAIL can, besides introducing a holistic approach, also provide health administrators with useful information about distribution of patient categories, e.g., for allocation of resources, Furthermore, researchers can gain access to all data. It is in this case essential to acknowledge that data originates from a clinical environment, thus the first years of implementation may be subject to issues of inadequate or erroneous data registration.

Different users may vary in what they consider most relevant and which indicators they believe can best answer their questions. The primary user group is healthcare personnel and administrators, who can use the indicator set as a supportive tool to ensure and monitor the delivery of holistic, interdisciplinary care for older patients living with frailty and to benchmark their results against fellow healthcare providers in Denmark. Identifying unwarranted variation can inform the refinement and development of national guidelines for the care of older people with frailty.

The underlying construct currently measured in DANFRAIL is in line with the original purpose of the database: Identifying unwanted variation and improving the quality of care for older people living with frailty. The indicator set is intended to be a balanced reflection of the CGA approach (Fig. 2)—bearing in mind that some important domains such as continuity of care and pharmacological review are not yet represented.

### Limitations

Using the DANFRAIL indicator set raises concerns of possible unintended consequences.

Data used by different stakeholders for different purposes may lead to potentially unintended consequences and conflicts. A major concern raised by clinicians and patients is the risk of frailism [36, 37]. Using the DANFRAIL dataset to facilitate discriminatory, prejudiced, or biased decisions based on frailty status stands in direct opposition to the database’s intended purpose. It is therefore crucial to acknowledge the risk of such misuse, and to actively implement measures to counteract it. However, this concern is not more critical than the existing challenges of ageism, which have also been documented [38, 39]. The steering committee is committed to addressing this issue proactively whenever it arises.

Not all CGA domains are currently represented, which constitutes a limitation of the construct of interest. In future development, domains such as pharmacological review (e.g., reducing polypharmacy and adverse effects of inappropriate combinations of medications) and basic needs will be included. Furthermore, measures capturing patients’ perspective on involvement and continuity of care—domains emphasized as particularly important by the representatives of relatives—could not be included due to limitations in existing data sources and data infrastructure in Denmark. Consequently, the primary aim of the database is not yet fully achieved, as data collection in this initial phase is restricted to acute hospital admissions and does not encompass all relevant age groups, care settings, or the everyday experiences of older people’s interaction with the healthcare system.

To address concerns from various stakeholders during the development process, the indicator set was adjusted. A major concern about increased workload and changes to work processes was raised by leaders of the Emergency Departments in Denmark. To allow for a smooth implementation process, the implementation period was extended and only patients staying at the hospital for at least 4 h will be included for data analyses.

The time required for data registration at the expense of clinical work was also a major concern expressed by several other stakeholders during the public consultation and is viewed as a threat to achieving high data completeness. The understanding of frailty as a risk factor of negative health outcomes is not routine in many specialties and professions. Educating and implementing this concept will require time, leadership, and effort. However, with the ongoing transformation of the healthcare agenda – emphasizing healthcare sustainability, integrated care models, patient-centered care, preventive care, and evidence-based practices,—these efforts are considered an important step toward fostering a shared understanding of a high-risk, high healthcare utilizing population. On average, CFS takes 44 s to perform [27, 40].

### Perspectives

Based on initial feedback from healthcare authorities, hospital boards and department leaderships, DANFRAIL has been positively received. It is expected to play a valuable role in the future development of quality in treating and caring for patients living with frailty. However, the steering committee has identified several important areas for further development:

- *Patient-relative’s perspective*: to accommodate the patient-relative's perspective on quality of care, a greater attention to gather qualitative data in a structured and unbiased format is needed. The Danish National Survey of Patient Experiences (LUP) is an annual nationwide survey that collects feedback from patients about their experiences with acute and non-acute hospitals contacts in Denmark. It aims to support quality improvement by highlighting strengths and identifying areas for development from the patient’s perspective. These data may provide some of the insights needed and will be explored in future developmental work within the steering committee.

*- Broader population*: including a wider population by lowering the age to, e.g., 70 years and/or including additional healthcare areas such as acute admissions to non-emergency departments, planned admissions, and outpatient care at hospitals.

- *Continuity in care*: monitoring care throughout the entire patient pathway, rather than being limited to hospital settings. The database will actively seek more collaboration and inclusion of data from primary care (municipal services and primary care physicians) as soon as this becomes technically possible.

*- Frailty research*: in addition to improving quality of care, the database holds significant research potential, providing baseline nationwide data and information on the continuity of acute care for all older patients, both with and without frailty. Since older people living with frailty are often excluded from clinical research [41, 42], DANFRAIL can provide valuable real-world evidence to address important research questions.

## Conclusion

A national Danish quality database focusing on older patients living with frailty is being implemented across all Danish hospitals during 2024–2025, with the aim of improving care quality and reducing unwarranted variation. Using the CGA framework, the DANFRAIL indicator set comprises six process and two result indicators, designed to identify high-risk patients and support preventive efforts to reduce or mitigate the consequences of frailty. Continuous education, development of technical solutions, and strong emphasis on person-centeredness and continuity of care are essential to ensure improvements in the care of older people living with frailty, regardless of where they encounter the healthcare system.

## Data Availability

The data that supports the findings of this study are available at https://www.sundk.dk (in Danish).
